# Atypical Enteropathogenic *Escherichia coli* Secretes Plasmid Encoded Toxin

**DOI:** 10.1155/2014/896235

**Published:** 2014-05-11

**Authors:** Rita C. Ruiz, Keyde C. M. Melo, Sarita S. Rossato, Camila M. Barbosa, Lívia M. Corrêa, Waldir P. Elias, Roxane M. F. Piazza

**Affiliations:** Laboratório de Bacteriologia, Instituto Butantan, Avenida Vital Brazil 1500, 05503-900 São Paulo, SP, Brazil

## Abstract

Plasmid encoded toxin (Pet) is a serine protease originally described in enteroaggregative *Escherichia coli* (EAEC) prototype strain 042 whose entire characterization was essentially obtained from studies performed with the purified toxin. Here we show that Pet is not exclusive to EAEC. Atypical enteropathogenic *Escherichia coli* (aEPEC) strains, isolated from diarrhea cases, express Pet and its detection in supernatants of infected HEp-2 cells coincides with the appearance of cell damage, which, in turn, were similar to those described with purified Pet. Pet secretion and the cytotoxic effects are time and culture medium dependent. In presence of DMEM supplemented with tryptone cell rounding and detachment were observed after just 5 h of incubation with the bacteria. In the absence of tryptone, the cytotoxic effects were detected only after 24 h of infection. We also show that, in addition to the prototype EAEC, other *pet*+ EAEC strains, also isolated from diarrhea cases, induce cellular damage in the same degree as the aEPEC. The cytotoxic effects of EAEC and aEPEC strains were significantly reduced in the presence of a serine protease inhibitor or anti-Pet IgG serum. Our results show a common aspect between the aEPEC and EAEC and provide the first evidence pointing to a role of Pet in aEPEC pathogenesis.

## 1. Introduction

Most bacterial enterotoxins, with a few exceptions, are proteases known to catalyze the hydrolytic cleavage of proteins and peptides in order to improve nutrient uptake by the bacteria [1]. As a consequence, many of these proteases are involved in the pathogenic processes associated with a number of infections and are, thus, considered important virulence factors [2, 3]. For instance, members of the family of the serine protease autotransporters of the Enterobacteriaceae (SPATE) have been associated with pathogenic strains and are among the virulence proteins predominantly secreted by the pathogens of this family [4].

The plasmid encoded toxin (Pet), so far described only in enteroaggregative* Escherichia coli* (EAEC), is certainly the best studied member of the SPATE family [5–7]. This toxin, an autotransporter prototype, is a 104 kDa protein that enters the cell via clathrin-coated vesicles. It reaches the Golgi complex and then the rough endoplasmic reticulum through the retrograde movement [8], where it disrupts *α*-fodrin activity, leading to a loss of actin stress fibers, cytoskeleton contraction and, finally, to cell rounding and detachment [9]. In addition cytokeratin 8 has recently been described as an important receptor for Pet in epithelial cells [10].

EAEC, defined by its aggregating pattern of adherence to epithelial cells [11], has been associated with persistent diarrhea in children from both developing and industrialized countries, as well as traveler's diarrhea [12]. Other than the binding of EAEC to the epithelial cell, it is known that this bacterium expresses enterotoxins and cytotoxins, including the Pet toxin, which leads to a secretory diarrhea and mucosal inflammation [13].

Atypical enteropathogenic* Escherichia coli* (aEPEC) have been associated with both acute childhood diarrhea [14–16] and persistent diarrhea [17, 18]. However, their pathogenicity is controversial since aEPEC have also been found in diarrheic and nondiarrheic patients. On the other hand, several studies have shown that aEPEC strains present high heterogeneity with frequent occurrence of virulence factors previously described in other diarrheagenic* E. coli* (DEC) pathotypes [19–21]. As a result, these strains can express different combinations of virulence factors, which may explain the isolation of aEPEC from both diarrheic and nondiarrheic individuals [22].

The presence of genes encoding virulence factors in plasmids, PAIs, transposons, or bacteriophages has allowed genetic recombination that may contribute to pathogenesis. Recently, SPATE-encoding genes from diarrheagenic* E. coli* (DEC) were found in aEPEC strains through the amplification of small fragments of the gene [23].

The expression of enterotoxins and cytotoxins has been shown to be determinant in the pathogenic processes caused by several enteric bacteria. However the global mechanism of diarrhea caused by aEPEC, which includes ion loss to the intestinal lumen and the secretion of enterotoxins, is poorly understood. For these reasons, we investigated the expression of Pet in aEPEC strains isolated from diarrhea cases [22]. In this work we show, for the first time, that toxin Pet is not exclusive to EAEC and that aEPEC also secretes Pet which induces cytotoxic effects similar to those observed in experiments with the purified toxin, on which the majority of the published studies on Pet toxin are based [8, 9, 24–26].

Our results point to the horizontal transfer of genes encoding the autotransporter protein, which can be an important factor in the emergence of highly virulent strains. We also show that EAEC strains, also obtained from diarrhea cases [27], induce cytotoxic effects similar to those observed with the EAEC prototype 042 [28], suggesting that the expression of Pet correlates with pathogenesis.

## 2. Materials and Methods

### 2.1. Bacterial Strains and Culture Conditions

For this study we selected four aEPEC (Table 1) and three EAEC strains (40 A5, 215 A3, and 252 A2) harboring the* pet* gene, isolated from cases of acute diarrhea [22, 23, 27]. One aEPEC strain lacking the* pet* gene was included as negative control [23].

The EAEC 042 [29] and Pet purified (200 *μ*g/mL) [8, 9] were used as positive controls and* E. coli* HB101 strain was used as a negative control [30].

Bacteria were cultivated in Luria-Bertani (LB) (Gibco, Rockville, MD) agar or LB broth before each experiment. For the cytotoxicity assays, 100 *μ*L bacterial culture was seeded in 3 mL DMEM (Gibco, Rockville, MD) or DMEM containing tryptone 1% (DMEM-tryptone) and cultured at 37°C with shaking at 200 rpm until the exponential phase.

### 2.2. Cell Culture

HEp-2 cells (ATCC N° CCL-23) were grown in DMEM (Gibco, Rockville, MD) supplemented with 10% fetal bovine serum (FBS) (Cultilab, Campinas, São Paulo) in a humidified atmosphere with 5% CO_2_, at 37°C. The subcultures were harvested with 10 mM EDTA and 0.25% trypsin in PBS, pH 7.4. For experimental use, the trypsinized cells were seeded, at 2 × 10^5^ cells/well, in 24-well culture plates containing 13 mm round cover glasses. The cells were then submitted to cytotoxicity assays.

### 2.3. Detection of Pet Expression by Immunoblotting

The expression of Pet was verified by immunoblotting performed with the IgG enriched fraction of a rabbit polyclonal anti-Pet serum (anti-Pet IgG) [27]. Briefly, aliquots of concentrated aEPEC culture supernatant containing 10 *μ*g of protein were loaded in each well in a 12% SDS-polyacrylamide gel [31, 32]. After electrophoresis, the separated proteins were transferred to a nitrocellulose membrane (Hybond C-Extra, Amersham Life Science) at 350 mA for 1 h at 4°C.

After the transfer and blocking stages, the membranes were probed with 200 *μ*g/mL of anti-Pet IgG for 1 h at room temperature. The membrane was then washed and incubated for 1 h with anti-rabbit IgG diluted 1 : 5.000 and analyzed by either Diaminobenzidine (DAB) (Sigma-Aldrich, St. Louis, MO) or ECL chemiluminescence system (Amersham Biosciences), according to the manufacturer's instructions.

### 2.4. Cytotoxicity Assays

Cell cultures were infected, in the presence of 10% FBS, at an MOI of 10 with the different bacterial strains cultivated in DMEM or DMEM-tryptone, and then incubated for 5 or 24 h, respectively, at 37°C.

To evaluate the cytotoxic effects, the cells were washed twice with PBS, fixed with 70% methanol, and stained with 10% Giemsa (Sigma-Aldrich, St. Louis, MO). The parameters used for the cytotoxicity evaluation were based on the criteria defined by Saidi and Sears [33] and Navarro-García et al. [24] where a score of 1+ indicates the presence of elongated or rounded cells greater than those observed in the control (but with less than 50% of cells affected); 2+ indicates that more than 50% of the cells are rounded but detachment was less than 50%; 3+ indicates that more than 50% of the cells are detached and all remaining cells are rounded; and 4+ indicates that all (or nearly all) cells are detached from the glass. Purified Pet, from clone pCEFN1 [34], at a concentration of 200 *μ*g/mL, and the prototype EAEC 042 were used as positive controls, while strain aEPEC BA 3170 (*pet*−) and* E. coli* HB101 served as negative controls.

In order to confirm the expression of Pet in this system, the culture medium was removed and precipitated with trichloroacetic acid (TCA). The precipitate was then submitted to SDS/PAGE and immunodetection of the toxin was performed with anti-Pet IgG, developed with DAB.

### 2.5. Neutralization of the Cytotoxic Effects

The neutralization test was performed using anti-Pet IgG [27] or the serine protease inhibitor phenylmethylsulfonyl fluoride (PMSF) (Boehringer, Indianapolis, IN), as described in Navarro-García et al. [24]. The different aEPEC strains were grown in DMEM or DMEM-tryptone, at 37°C, at 200 rpm until reaching OD 0.8. Next, 20 *μ*L of each culture was preincubated with anti-Pet IgG at the concentrations of 125 *μ*g/mL and 250 *μ*g/mL for 30 min at 37°C. Alternatively, the same cultures were preincubated with PMSF at the concentrations of 0.3 mM, 0.6 mM, and 1.25 mM for 15 min at 37°C. The preincubated aliquots were then added to the cells in fresh medium and incubated for 5 h at 37°C prior to standard fixation and staining. The positive controls were the prototype EAEC 042 and Pet (200 *μ*g/mL) preincubated with the anti-Pet IgG or PMSF. Strains cultivated with the culture medium only were also used as controls. All experiments were performed at least three times, each time in triplicate.

### 2.6. Statistical Analyses

All data were analyzed using GraphPad Prism 5.0 (San Diego, CA). For use in nonparametric analyses, the Friedman with Dunn's multiple comparison test was performed. In parametric analyses, the 1-way ANOVA with Tukey's multiple comparison test was carried out. Shapiro-Wilk normality test was used for all data. Results were considered statistically significant when *P* < 0.05.

## 3. Results

### 3.1. Reactivity of Polyclonal Anti-Pet Antiserum with aEPEC Strains

**Figure 1:**
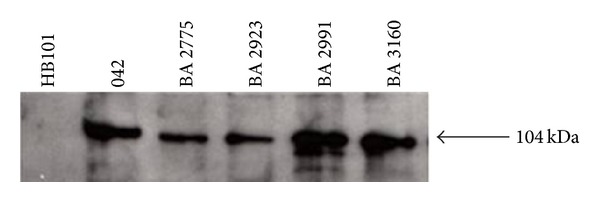
Detection of Pet in culture supernatants of* pet*+ aEPEC strains. The bacterial supernatants, cultivated in LB broth, were precipitated with TCA. Negative control* E. coli* sample HB101. Positive control EAEC 042. aEPEC strains BA 2775, BA 2923,BA 2991, and BA 3160.

The* pet* gene has been identified in aEPEC strains [23]. But it does not necessarily mean that the toxin is expressed, and up to the present date, aEPEC strains have not been shown to actually express Pet. For this reason we initially checked Pet secretion in culture supernatants of* pet*+ aEPEC strains. Culture supernatants were precipitated with TCA and subjected to immunoblotting with anti-Pet IgG [27]. A 104 kDa band, the molecular weight of Pet, was detected in all four aEPEC strains, but not in* E. coli* strain HB101, used as a negative control (Figure 1), strongly suggesting that Pet is secreted by these strains.

### 3.2. Cytotoxic Effects Induced by *pet*+ Bacterial Strains

Since Pet is apparently secreted by these aEPEC strains, we next verified whether it could be associated with cellular damage caused by them.

The secretion of Pet was initially achieved with cultures in LB broth. However, LB broth is not the ideal medium for the study of bacteria-host cell interactions. Betancourt-Sanchez and Navarro-Garcia [28] showed that DMEM, a culture medium used for both eukaryotic and bacterial cells, with the addition of tryptone, which is present in LB broth, improves the secretion of Pet by strain EAEC 042 [28].

In this work we studied the cytotoxic effects of Pet by incubating the aEPEC strains grown in DMEM or DMEM-tryptone with HEp-2 cells for 24 and 5 h, respectively. The cytotoxic effects were analyzed through light microscopy observation and quantification of postinfection adhered cells. Cells incubated with the same culture medium, without infection, were used as negative controls and presented 100% of viable adherent cells.

In parallel, the EAEC strains, which also carry the* pet* gene [27], were assayed in order to compare the cytotoxic effects of both diarrheagenic* E. coli* pathotypes.

**Figure 2:**
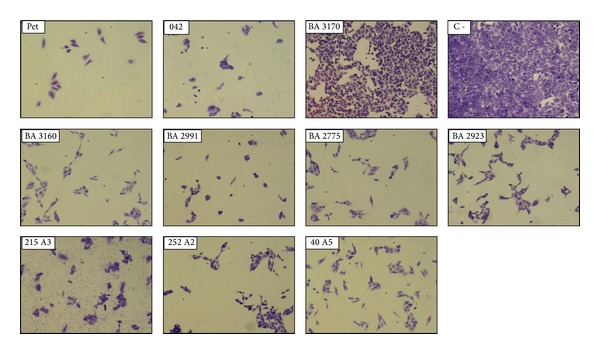
Light microscopy of HEp-2 cells incubated with different EPEC and EAEC strains in DMEM. After 24 h of incubation the cells were washed, fixed, and stained with Giemsa. The positive controls for this experiment were the EAEC prototype 042 and Pet at 200 *μ*g/mL. Magnification: 100x.

**Figure 3:**
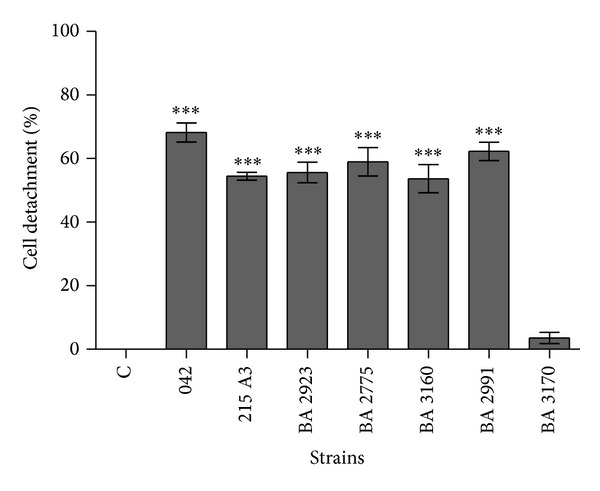
Percentage of HEp-2 cells detachment after 5 h of incubation with the* pet*+ aEPEC strains BA 3160; BA 2991; BA 2923; BA 2775 and the EAEC strain 215 A3 in the presence of DMEM-tryptone. Positive control EAEC 042. Negative controls just the cells (C) and* pet*− aEPEC strain BA 3170. The data refer to the mean values of four independent experiments. *Significantly different when compared to the negative control (C). One-way analysis of variance (ANOVA) with Tukey's test was carried out to determine statistically significant differences from untreated controls. ***P < 0.001.

**Figure 4:**
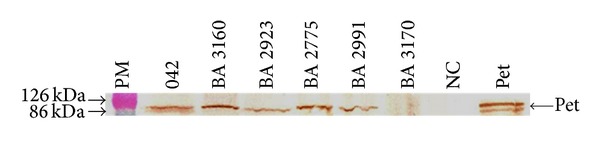
Pet is present in the HEp-2 culture supernatant after 5 h of incubation in DMEM-tryptone with the aEPEC strains BA 3160, BA 2923, BA 2775, and BA 2991, as detected with anti-Pet IgG and developed with DAB. MW: molecular weight. Positive controls: EAEC 042 and Pet. Negative controls: aEPEC BA 3170 and noninfected cells (NC).

In the absence of tryptone, both* pet*+ aEPEC and* pet*+ EAEC strains induced cell rounding and detachment after 24 h of incubation (Figure 2). But no cytotoxic effect could be observed sooner than that. When tryptone was added to DMEM, cell detachment was observed after only 5 h of incubation (Figure 3), coinciding with the detection of Pet in the culture supernatant of the infected HEp-2 cells (Figure 4). These results are in agreement with Betancourt-Sanchez and Navarro-Garcia [28], who showed that the addition of tryptone to DMEM anticipates the secretion of Pet, which can then be detected after 5 h of incubation, while in the absence of tryptone the secretion of Pet by strain EAEC 042 could only be detected after 18 h of incubation [28].

Since the growth kinetics of EAEC and aEPEC are similar in both DMEM and DMEM-tryptone (data not shown), the cytotoxic effects induced by these strains could be compared after any given incubation time. We show here that aEPEC clearly induces cell rounding and detachment in the same degree as EAEC, or even more.

### 3.3. Neutralization of the Cytotoxic Effect

**Figure 5:**
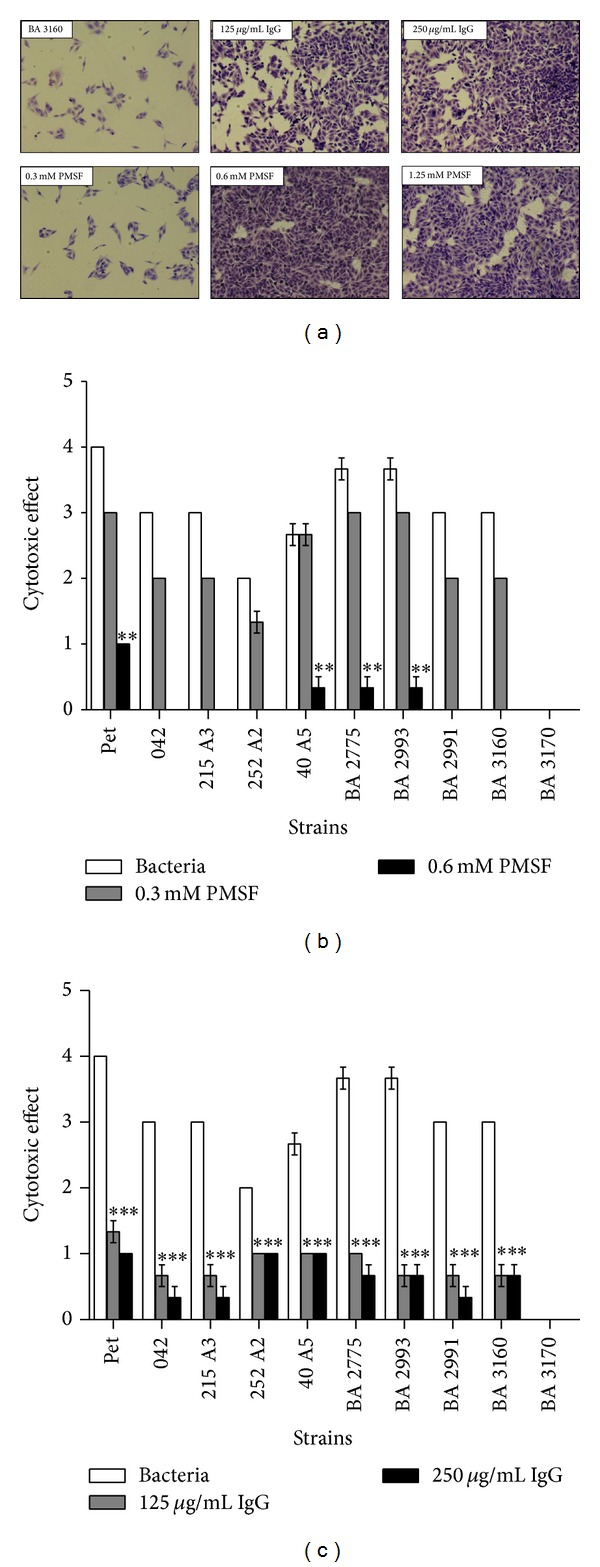
Kinetics of the neutralization of the cytotoxic effects on HEp-2 cells after previous incubation of the bacterial strains with anti-Pet IgG (125 *μ*g/mL and 250 *μ*g/mL) or with PMSF at the concentrations of 0.3, 0.6, and 1.25 mM. Light microscopy of the cells incubated with aEPEC strain BA 3160; magnification: 100x (a). Cytotoxic effect on cells incubated with aEPEC strains BA 2775, BA 3160, BA 2923, and BA 2991 and EAEC strains 40 A5, 215 A3, and 252 A2. Positive controls were EAEC 042 and Pet. Negative controls were aEPEC BA 3170 and cells alone incubated with PMSF. *Statistically significant difference when compared to the control (bacteria) as determined using the Friedman with Dunn nonparametric test, **P < 0.01 (b) or incubated with the anti-Pet IgG. *Statistically significant difference when compared to the control (bacteria) as determined using one-way analysis of variance (ANOVA) with Tukey's test, ***P < 0.001 (c). The results refer to the mean values of three independent experiments performed in triplicate.

To verify whether the cytotoxic effects caused by the aEPEC and EAEC strains were induced by a serine protease, the bacteria were incubated, before the infection pulse, with either 0.3 mM, 0.6 mM, or 1.25 mM PMSF, a serine protease inhibitor. The most effective PMSF concentration was 0.6 mM with both aEPEC and EAEC pathotypes (Figures 5(a) and 5(b)). The cytotoxic effects were also significantly reduced when the strains were previously incubated with anti-Pet IgG at both concentrations of 125 *μ*g/mL and 250 *μ*g/mL, as compared to the cultures infected with untreated bacteria (Figure 5(c)). Neither the preimmune serum nor nonspecific IgG fractions induced any cytotoxic effects (data not shown). Together, these results show that Pet is the toxin responsible for the observed cytotoxicity.

## 4. Discussion

SPATEs, which comprise a large group of trypsin-like serine proteases, promote their own secretion through the type V secretion system and have been described as important virulence factors in the pathogenic processes caused by* Shigella* spp., uropathogenic* E. coli*, and DEC [5]. Pet, studied here, was described and characterized only in 042 EAEC prototype strain [5–7]. This toxin is known to be cytotoxic to cultured epithelial cells, such as Caco-2, HT29, HEp-2, and CHO [24, 35]. It causes cytoskeletal rearrangements and contraction and release of the cellular focal contacts in cell monolayers, indicating that it is an important virulence factor in the pathogenesis of EAEC infection [24–26]. However, practically all the knowledge on this toxin was obtained with the purified protein that, at a concentration of 37 *μ*g/mL, causes irreversible cell damage [8, 9, 24–26].

Here we investigated the secretion of Pet in aEPEC strains harboring the* pet* gene [23] as well as its cytotoxic potential through the analysis in the context of the direct action of the bacteria on cultured cells. In addition, we studied the cytotoxicity of three different* pet*+ EAEC strains, not yet been demonstrated. The expression of Pet has only been described in the EAEC prototype strain 042 [27], which was used here as a positive control.

Gene expression may be influenced by a range of environmental stimuli, including the composition of the culture medium and host cell contact [24, 27, 28]. For instance, when DMEM is supplemented with tryptone it induces a positive upregulation of Pet mRNA transcription. Tryptone is a constituent of LB broth, which normally favors Pet expression [28]. Therefore, we investigated cytotoxicity of* pet*+ aEPEC and EAEC strains in a comparative study using DMEM both containing tryptone and not.

We show that the induction of the cytotoxic effects by both* E. coli* pathotypes is time and culture medium dependent. These findings are in agreement with Betancourt-Sanchez and Navarro-Garcia [28], who demonstrated that Pet secretion by EAEC 042 is anticipated when tryptone was added to the eukaryotic cell culture medium.

In this study, the cytotoxic effects induced by both aEPEC and EAEC strains were equally intense and were observed as soon as the toxin was detected in the supernatant of the cell cultures.

Adhesins have been suggested to be important in the process of toxin Pet delivery by EAEC based on the fact that strain HB101, which does not express adhesins but is a Pet hyperproducer (HB101-Pet), could not deliver Pet to epithelial cells, as opposed to the observation with EAEC 042, suggesting that adhesion of EAEC to the cell is important for proper toxin delivery [28].

However, the aEPEC strains studied here were classified, based on adhesion assays, as nonadherent to epithelial cells [36]. Although the results obtained with HB101-Pet [28] and aEPEC may seem contradictory, aEPEC, as opposed to HB101-Pet, is pathogenic, having been isolated from diarrheic patients, and expresses several additional virulence factors, such as adhesins* cah*,* ehaA*,* ehaC*, and* espI* [23]. Furthermore, the classic adhesion assays are designed to define the adhesion pattern of the bacteria to the cell after a given incubation time. In the case of the aEPEC pathotype, six hours were necessary for the adhesion pattern to be observed [37]. Therefore, this assay may not reflect possible bacteria-cell interactions that might occur in a shorter incubation time and that could be enough for the delivery of the toxin to the cell. Pet was detected inside the cells after only 30 min of incubation with purified toxin [26, 38]. For instance, studies performed with the purified toxin show that Pet binds to the cell surface on its own and is endocytosed by clathrin-coated vesicles, suggesting the existence of an internalization process which is independent of bacterial adhesion to the cell [26].

To establish a correlation between the cellular damage observed in this work and toxin Pet, we preincubated the bacteria with either the serine protease inhibitor PMSF or the rabbit anti-Pet IgG enriched fraction. Both treatments considerably reduced or even completely prevented the damage induced to the cells. These results show that the cytotoxic effect induced by both DEC categories is due to toxin Pet.

The presence of genes encoding virulence factors in mobile units, such as plasmids, favors the horizontal transmission of these factors between bacteria and could explain the presence of the* pet* gene in aEPEC and its high heterogeneity [20, 21, 39]. In the EAEC prototype 042 strain a correlation between the presence of the aggregative adherence fimbriae II (AAF/II) and Pet, both located in the virulence plasmid (pAA), has been described [34]. Such correlation was not observed in the aEPEC strains in which we have demonstrated the expression of Pet. In these strains, only genes encoding adhesins* cah*,* ehaA*,* ehaC*, and* espI* could be detected, but not AAF/II encoding genes (unpublished data) or other SPATE genes [23]. In addition, these aEPEC strains belong to nonclassical serogroups known to be more diverse and in which many virulence genes have already been described, but whose expression is still poorly studied [39].

An additional interesting aspect that relates the SPATEs and the DEC pathotypes is that EAEC and typical enteropathogenic* Escherichia coli* (tEPEC) express different serine proteases [6, 40]. EAEC express Pet and Pic, while tEPEC express the* E. coli* secreted protein C (EspC) [41]. EspC was detected in the supernatant of cell cultures infected with the tEPEC prototype E2348/69 [42]. Like Pet, EspC is also internalized; it cleaves *α*-fodrin and alters the actin fibers of the cytoskeleton, inducing similar damage [43]. However the interaction of these toxins with the host is different, suggesting different roles in pathogenesis [44]. Interestingly all aEPEC* pet*+ studied here lack the* espC* gene sequence as well as other SPATE encoding genes [23].

To better understand the processes involving SPATEs, which present such diverse activities, they should be characterized in the context of each pathogen that expresses them. In the case of aEPEC, which does not present the* espC* gene and whose single pathogenic mechanism described so far is the attaching and effacing lesion, Pet might play an important role in cytotoxicity.

Both EAEC and aEPEC are known to present a high heterogeneity of virulence factors and have been isolated from both diarrheic and nondiarrheic patients. Afset et al. [17] and Nguyen et al. [18] demonstrated that, like EAEC, which is a pathotype classically described as an agent of persistent and traveler's diarrhea affecting individuals of all ages, aEPEC can also cause persistent diarrhea and affect individuals of all ages, as opposed to the tEPEC [21].

Though the* pet* gene was previously identified in aEPEC through the amplification of a fragment of 302 bp [23], we show here for the first time that Pet is indeed secreted by aEPEC and that it induces cell damage similar to those previously observed with EAEC. This is the first evidence pointing to a role of Pet in aEPEC pathogenesis. These data show a common aspect between the aEPEC and EAEC strains, which is apparently important in the pathogenesis, since all bacterial strains used here were isolated from cases of diarrhea [22, 27]. Determining the location of the* pet* gene, the importance of bacterial adherence for toxin delivery, and the contribution of Pet to aEPEC pathogenicity are the goals of our next studies.

## Figures and Tables

**Table 1:** Characteristics of the aEPEC strains.

Strains	Serotype	SPATE virulence genes
BA 2923	O34:H6	*pet, cah, ehaA, ehaC, espI *
BA 2991	O34:H−	*pet, cah, ehaA, ehaC, espI *
BA 2775	O113:H19	*pet, ehaA *
BA 3160	O110:H−	*pet, ehaA *
BA 3170	O145:H2	*espC *
